# COVLIAS 1.0_Lesion_ vs. MedSeg: An Artificial Intelligence Framework for Automated Lesion Segmentation in COVID-19 Lung Computed Tomography Scans

**DOI:** 10.3390/diagnostics12051283

**Published:** 2022-05-21

**Authors:** Jasjit S. Suri, Sushant Agarwal, Gian Luca Chabert, Alessandro Carriero, Alessio Paschè, Pietro S. C. Danna, Luca Saba, Armin Mehmedović, Gavino Faa, Inder M. Singh, Monika Turk, Paramjit S. Chadha, Amer M. Johri, Narendra N. Khanna, Sophie Mavrogeni, John R. Laird, Gyan Pareek, Martin Miner, David W. Sobel, Antonella Balestrieri, Petros P. Sfikakis, George Tsoulfas, Athanasios D. Protogerou, Durga Prasanna Misra, Vikas Agarwal, George D. Kitas, Jagjit S. Teji, Mustafa Al-Maini, Surinder K. Dhanjil, Andrew Nicolaides, Aditya Sharma, Vijay Rathore, Mostafa Fatemi, Azra Alizad, Pudukode R. Krishnan, Ferenc Nagy, Zoltan Ruzsa, Mostafa M. Fouda, Subbaram Naidu, Klaudija Viskovic, Manudeep K. Kalra

**Affiliations:** 1 Stroke Diagnostic and Monitoring Division, AtheroPoint™, Roseville, CA 95661, USA; drindersingh1@gmail.com (I.M.S.); pomchadha@gmail.com (P.S.C.); 2 Advanced Knowledge Engineering Centre, GBTI, Roseville, CA 95661, USA; sushant.ag09@gmail.com; 3 Department of Computer Science Engineering, PSIT, Kanpur 209305, India; 4 Department of Radiology, Azienda Ospedaliero Universitaria (A.O.U.), 09124 Cagliari, Italy; gianchab@yahoo.com (G.L.C.); pascheale@gmail.com (A.P.); psc.dnn@gmail.com (P.S.C.D.); lucasabamd@gmail.com (L.S.); antonellabalestrieri@hotmail.com (A.B.); 5 Department of Radiology, “Maggiore della Carità” Hospital, University of Piemonte Orientale (UPO), Via Solaroli 17, 28100 Novara, Italy; profcarriero@virgilio.it; 6 University Hospital for Infectious Diseases, 10000 Zagreb, Croatia; mehmedovic.armin302@gmail.com (A.M.); klaudija.viskovic@bfm.hr (K.V.); 7 Department of Pathology, Azienda Ospedaliero Universitaria (A.O.U.), 09124 Cagliari, Italy; gavinofaa@gmail.com; 8 The Hanse-Wissenschaftskolleg Institute for Advanced Study, 27753 Delmenhorst, Germany; monika.turk84@gmail.com; 9 Department of Medicine, Division of Cardiology, Queen’s University, Kingston, ON K7L 3N6, Canada; johria@queensu.ca; 10 Department of Cardiology, Indraprastha APOLLO Hospitals, New Delhi 110076, India; drnnkhanna@gmail.com; 11 Cardiology Clinic, Onassis Cardiac Surgery Center, 17674 Athens, Greece; soma13@otenet.gr; 12 Heart and Vascular Institute, Adventist Health St. Helena, St Helena, CA 94574, USA; lairdjr@ah.org; 13 Minimally Invasive Urology Institute, Brown University, Providence, RI 02912, USA; gyan_pareek@brown.edu (G.P.); dwsobel@gmail.com (D.W.S.); 14 Men’s Health Center, Miriam Hospital, Providence, RI 02906, USA; martin_miner@brown.edu; 15 Rheumatology Unit, National Kapodistrian University of Athens, 15772 Athens, Greece; psfikakis@med.uoa.gr; 16 Department of Surgery, Aristoteleion University of Thessaloniki, 54124 Thessaloniki, Greece; tsoulfasg@gmail.com; 17 Cardiovascular Prevention and Research Unit, Department of Pathophysiology, National & Kapodistrian University of Athens, 15772 Athens, Greece; aprotog@med.uoa.gr; 18 Department of Immunology, Sanjay Gandhi Postgraduate Institute of Medical Sciences, Lucknow 226014, India; durgapmisra@gmail.com (D.P.M.); vikasagr@yahoo.com (V.A.); 19 Academic Affairs, Dudley Group NHS Foundation Trust, Dudley DY1 2HQ, UK; george.kitas@nhs.net; 20 Arthritis Research UK Epidemiology Unit, Manchester University, Manchester M13 9PL, UK; 21 Ann and Robert H. Lurie Children’s Hospital of Chicago, Chicago, IL 60611, USA; jsteji1@comcast.net; 22 Allergy, Clinical Immunology and Rheumatology Institute, Toronto, ON L4Z 4C4, Canada; almaini@hotmail.com; 23 AtheroPoint LLC, Roseville, CA 95661, USA; surinderdhanjil@gmail.com (S.K.D.); rajvivs888@gmail.com (V.R.); 24 Vascular Screening and Diagnostic Centre, University of Nicosia Medical School, Nicosia 2408, Cyprus; anicolaides1@gmail.com; 25 Division of Cardiovascular Medicine, University of Virginia, Charlottesville, VA 22908, USA; as8ah@hscmail.mcc.virginia.edu; 26 Department of Physiology and Biomedical Engineering, Mayo Clinic College of Medicine and Science, Rochester, MN 55905, USA; fatemi.mostafa@mayo.edu; 27 Department of Radiology, Mayo Clinic College of Medicine and Science, Rochester, MN 55905, USA; alizad.azra@mayo.edu; 28 Neurology Department, Fortis Hospital, Bangalore 560076, India; prkrish12@rediffmail.com; 29 Internal Medicine Department, University of Szeged, 6725 Szeged, Hungary; drnagytfer@hotmail.com; 30 Invasive Cardiology Division, University of Szeged, 6725 Szeged, Hungary; zruzsa@icloud.com; 31 Department of Electrical and Computer Engineering, Idaho State University, Pocatello, ID 83209, USA; mfouda@isu.edu; 32 Electrical Engineering Department, University of Minnesota, Duluth, MN 55812, USA; dsnaidu@d.umn.edu; 33 Department of Radiology, Massachusetts General Hospital, 55 Fruit Street, Boston, MA 02114, USA; mkalra@mgh.harvard.edu

**Keywords:** COVID-19, computed tomography, COVID lesions, ground-glass opacities, segmentation, hybrid deep learning

## Abstract

Background: COVID-19 is a disease with multiple variants, and is quickly spreading throughout the world. It is crucial to identify patients who are suspected of having COVID-19 early, because the vaccine is not readily available in certain parts of the world. Methodology: Lung computed tomography (CT) imaging can be used to diagnose COVID-19 as an alternative to the RT-PCR test in some cases. The occurrence of ground-glass opacities in the lung region is a characteristic of COVID-19 in chest CT scans, and these are daunting to locate and segment manually. The proposed study consists of a combination of solo deep learning (DL) and hybrid DL (HDL) models to tackle the lesion location and segmentation more quickly. One DL and four HDL models—namely, PSPNet, VGG-SegNet, ResNet-SegNet, VGG-UNet, and ResNet-UNet—were trained by an expert radiologist. The training scheme adopted a fivefold cross-validation strategy on a cohort of 3000 images selected from a set of 40 COVID-19-positive individuals. Results: The proposed variability study uses tracings from two trained radiologists as part of the validation. Five artificial intelligence (AI) models were benchmarked against MedSeg. The best AI model, ResNet-UNet, was superior to MedSeg by 9% and 15% for Dice and Jaccard, respectively, when compared against MD 1, and by 4% and 8%, respectively, when compared against MD 2. Statistical tests—namely, the Mann–Whitney test, paired *t*-test, and Wilcoxon test—demonstrated its stability and reliability, with *p* < 0.0001. The online system for each slice was <1 s. Conclusions: The AI models reliably located and segmented COVID-19 lesions in CT scans. The COVLIAS 1.0_Lesion_ lesion locator passed the intervariability test.

## 1. Introduction

Severe acute respiratory syndrome coronavirus 2 (SARS-CoV-2) is an infectious disease that poses a concern to humans worldwide. The World Health Organization (WHO) proclaimed COVID-19 (the novel coronavirus disease) as a global pandemic on 11 March 2020. COVID-19 is a rapidly spreading illness worldwide, yet hospital resources are limited. As of 1 December 2021, COVID-19 had led to the infection of 260 million people and 5.2 million deaths worldwide [1]. COVID-19 has clearly shown to have several molecular pathways [2], leading to myocardial injury [3], diabetes [4], pulmonary embolism [5], and thrombosis [6]. Due to the lack of an effective vaccine or medication, early detection of COVID-19 is critical to saving many lives and safeguarding frontline workers. Most medical staff have become infected due to their frequent contact with patients, significantly aggravating the already dire healthcare situation.

The early detection of COVID-19 is critical to saving many lives and protecting frontline workers, due to the lack of an appropriate vaccination or therapy. RT-PCR, or “reverse transcription-polymerase chain reaction”, is one of the gold standards for the detection of COVID-19 [7,8]. Furthermore, since the RT-PCR test is slow—causing delays in report generation—and has low sensitivity [9], there is a need for better detection methods. However, imaging-based diagnosis, including ultrasound [10], chest X-ray [11], and chest computed tomography (CT) [12], is becoming more popular in detecting and managing infection with COVID-19 [13,14]. CT has demonstrated great sensitivity and repeatability in the diagnosis of COVID-19, and for body imaging in general [15]. It is a significant and trustworthy complement to RT-PCR testing in identifying the disease [16,17,18]. The main imaging advantage of CT [15,19,20] imaging is capturing anomalies such as ground-glass opacity (GGO) [21,22], consolidation, and other opacities seen in the CT of a COVID-19 patient [23]. The anomaly of GGO is a prevalent feature in most chest CT lung images [14,24,25,26]. Due to time constraints and the sheer volume of studies, most radiologists use a judgmental and semantic approach to evaluate the COVID-19 lesions with different opacities. Furthermore, the manual and semi-automated assessment is subjective, slow, and time-consuming [27,28,29,30]. As a result, rapid and error-free detection and real-time prognostic solutions are required for early COVID-19 illness to improve the speed of diagnosis.

Artificial intelligence (AI) has accelerated research and development in almost every field, including healthcare imaging [31,32,33]. The ability of AI techniques to replicate what is done manually has made detection and diagnosis of this disease faster [34,35,36,37,38,39,40,41,42,43,44,45,46]. The AI techniques try to accurately mimic the human brain using deep neural networks. This makes them suitable for solving medical imaging problems. Deep learning (DL) is an extension of AI that uses dense layers to deliver completely automatic feature extraction, classification, and segmentation [47,48,49,50,51,52,53].

**Figure 1:**
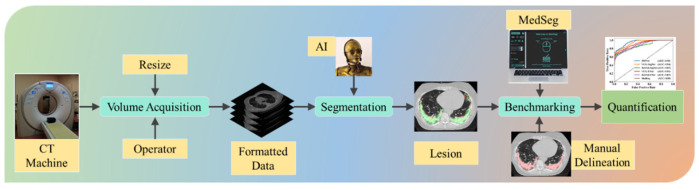
AI system workflow for comparing COVLIAS 1.0_Lesion_ against MedSeg.

DL has advantages, but it also has drawbacks and unknowns, such as optimization of the learning rate, determining the number of epochs, preventing overfitting, handling large datasets, and functioning in a multiresolution framework [54]. This is also known as hyperparameter tuning, which is the most crucial task when accurately training a DL model. Recently published studies by Suri et al. prove that using hybrid DL (HDL) models over solo DL [55,56] models in the medical domain can help to learn complex imaging features quickly and accurately [57,58,59]. Transfer learning can also be adapted for knowledge transfer from one model to another. This process helps train the DL models faster, and with fewer images [60,61]. The proposed study utilizes SDL and HDL models to segment COVID-19-based lesions in CT lung images. To prove the robustness of the AI systems, we postulate two conditions as the hypotheses: (a) the performance of the AI model benchmarked against two manual delineations must be within 10% of one another, and (b) the HDL model outperforms the SDL model in terms of performance. Figure 1 depicts the global COVLIAS 1.0_Lesion_ system for COVID-19-based lesion segmentation using AI models, consisting of volume acquisition, online segmentation, and benchmarking against MedSeg, along with performance evaluation.

The main contributions of this study are as follows: (1) The proposed study consists of a combination of solo DL and HDL to tackle the lesion location for faster segmentation. One DL and four HDL models—namely, PSPNet, VGG-SegNet, ResNet-SegNet, VGG-UNet, and ResNet-UNet—were trained by an expert radiologist. (2) The training scheme adopted a fivefold cross-validation strategy on a cohort of 3000 images selected from a set of 40 COVID-19-positive individuals. Performance evaluation was carried out using systems such as (a) Dice similarity, (b) Jaccard index, (c) Bland–Altman plots, and (d) regression plots. (3) COVLIAS 1.0_Lesion_ was benchmarked against the online MedSeg system, demonstrating COVLIAS 1.0_Lesion_ to be superior to MedSeg when compared against Manual Delineation 1 and Manual Delineation 2. (4) The proposed interobserver variability study used tracings from two trained radiologists as part of the validation. (5) Statistical tests—namely, the Mann–Whitney test, paired *t*-test, and Wilcoxon test—demonstrated its stability and reliability, along with the *p*-values. (6) The online system for each slice was <1 s.

The layout of this lesion segmentation study is as follows: In Section 2, we present the patient demographics and types of AI architectures. The results of the experimental protocol using the AI architectures, along with the performance evaluation, are shown in Section 3. The in-depth discussion is elaborated in Section 4, where we present our findings, benchmarking tables, strengths, weaknesses, and extensions of our study. The study concludes in Section 5.

## 2. Methods

### 2.1. Demographics and Baseline Characteristics

**Figure 2:**
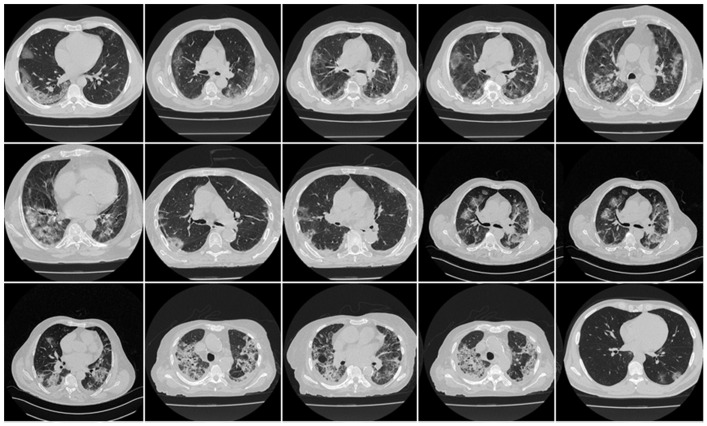
Raw CT images from the Croatia dataset.

Approximately 3000 CT images (collected from 40 patients from Croatia) were used to create the training cohort (Figure 2). The patients had a mean age of 66 (SD 7.988), with 35 males (71.4 %) and the remainder females. In the cohort, the average GGO and consolidation scores were 2 and 1.2, respectively. Out of the 40 patients who participated in this study, all had a cough, 85% had dyspnoea, 28% had hypertension, 14% were smokers, and none had a sore throat, diabetes, COPD, or cancer. None of them were admitted to the intensive care unit (ICU) or died due to COVID-19 infection.

### 2.2. Image Acquisition and Data Preparation

This proposed study used a Croatian cohort of 40 COVID-19-positive patients. The retrospective cohort study was conducted from 1 March to 31 December 2020, at the University Hospital for Infectious Diseases in Zagreb, Croatia. All patients over the age of 18 who agreed to participate in the study had a positive RT-PCR test for the SARS-CoV-2 virus, underwent thoracic MDCT during their hospital stay, and met at least one of the following criteria: hypoxia (oxygen saturation below 92%), tachypnea (respiratory rate above 22 per minute), tachycardia (pulse rate > 100), or hypotension (systolic blood pressure 100 mmHg) prior to starting the study. The UHID Ethics Committee gave their consent. The acquisition was carried out using a 64-detector scanner from FCT Speedia HD (from Fujifilm Corporation, Tokyo, Japan, 2017), while the acquisition protocol consisted of a single full inspiratory breath-hold for collection of CT scans of the thorax in the craniocaudal direction.

Researchers used Hitachi Ltd.’s (Tokyo, Japan) Whole-Body X-ray CT System with Supria Software, and a typical imaging method to view the images (System Software Version: V2.25, Copyright Hitachi, Ltd., 2017). When scanning, the following values were used: wide focus, 120 kV tube voltage, 350 mA tube current, and 0.75 s rotation speed in the IntelligentEC (automatic tube-current modulation) mode. We followed the standardized protocol for reconstruction as adopted in our previous studies where, for multi-recon options, the field of view was 350 mm, the slice thickness was 5 mm (0.625 × 64), and the table pitch was 1.3281. We selected a slice thickness of 1.25 mm and a recon index of 1 mm for picture filter 22 (lung standard) with the Intelli IP Lv.2 iterative algorithm (WW1600/WL600). Furthermore, for picture filter 31 (mediastinal), with the Lv.3 Intelli IP iterative algorithm (WW450/WL45), the slice thickness was 1.25 mm and the recon index was 1 mm.

**Figure 3:**
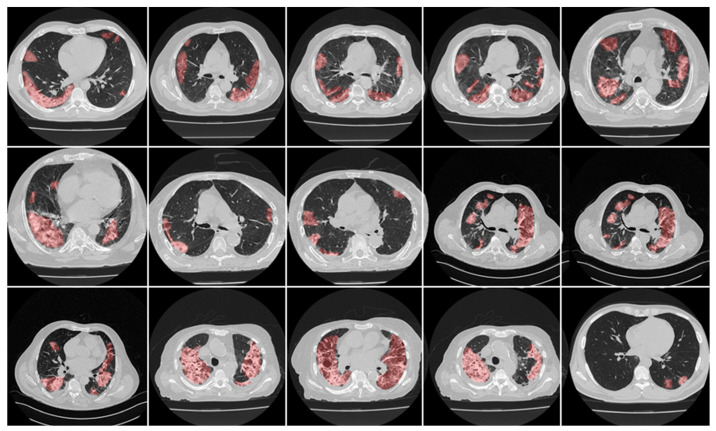
Manual delineation overlays (red) from tracer 1 on raw CT images.

**Figure 4:**
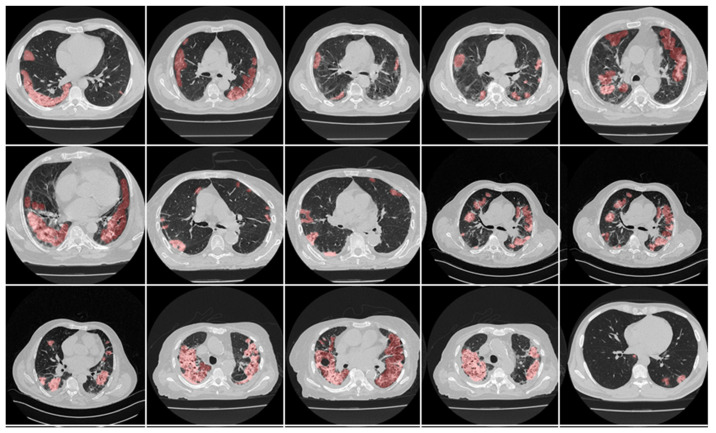
Manual delineation overlays (red) from tracer 2 on raw CT images.

Scanned areas were chosen based on the presence of no metallic objects and reasonable image quality without artefacts or blurriness caused by the movement of the patients during the conduction of the scan. Each patient’s CT volume in this cohort consisted of ~300 slices. The senior radiologist (K.V.) carefully selected ~70 CT slices (512 × 512 px^2^) that preserved most of the lung region (only accounting for about 20% of the total CT slices). Figure 3 and Figure 4 show the annotated lesions from tracers 1 and 2, respectively, in red, with the raw CT image as the background.

### 2.3. The Deep Learning Models

The proposed study consists of a combination of solo deep learning (DL) and hybrid DL (HDL) models to tackle the lesion location and lesion segmentation more quickly. It was recently shown that the combination of two DL models has more feature-extraction power compared to the solo DL models; this motivation brought the innovation of combining two solo DL models. This study therefore implemented four HDL models—namely, VGG-SegNet, ResNet-SegNet, VGG-UNet, and ResNet-UNet—that were trained by an expert radiologist. This was then also benchmarked against a solo DL model, namely, PSPNet.

By replacing the kernel filter in the initial layer with 11- and 5-sized filters, the VGGNet architecture was meant to reduce training time [62]. VGGNet was extremely efficient and speedy, but it had a problem in optimization due to vanishing gradients. During backpropagation, it resulted in training with substantially less or no weights, because it was multiplied by the gradient at each epoch, and the update to the initial layers was very modest. Residual Network, or ResNet [63], was created to address this issue. A new link called the “skip connection” was created in this architecture, allowing gradients to bypass a limited number of layers and thereby resolve the issue of the vanishing gradient problem. Furthermore, during the backpropagation step, another modification to the network—namely, an identity function—kept the local gradient value at a non-zero quantity.

The HDL models were designed by combining one DL (i.e., VGG or ResNet, in our study) with another DL (i.e., UNet or SegNet, in our study), thereby producing a superior network with the advantages of both parent networks. The VGG-SegNet, VGG-UNet, ResNet-SegNet, and ResNet-UNet architectures employed in this research are made up of three parts: an encoder, a decoder, and a pixel-wise softmax classifier. The details of the SDL and HDL models are discussed in the following sections.

#### 2.3.1. PSPNet—Solo DL Model

**Figure 5:**
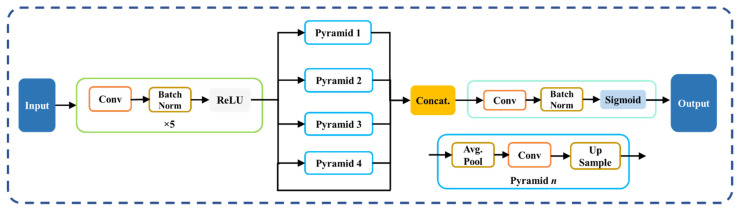
PSPNet’s architecture.

The pyramid scene parsing network (PSPNet) [64] is a semantic segmentation network that takes into account the image’s overall context. PSPNet includes four sections to its design (Figure 5): (1) input, (2) feature map, (3) pyramid pooling module, and (4) output [65,66]. The segmented image is sent into the network, which then uses a set of dilated convolution and pooling blocks to extract the feature map. The network’s heart is the pyramid pooling module, which helps capture the global context of the image/feature map constructed in the previous stage. This section is divided into four sections, each with its own scaling capabilities. This module’s scaling options are 1, 2, 3, and 6, with 1 × 1 scaling assisting in the acquisition of spatial data, and thereby increasing the resolution of the acquired features. The higher-resolution features are captured by the 6 × 6 scaling. All of the outputs from these four components are pooled at the end of this module using global average pooling. The global average pooling output is sent to a collection of convolutional layers in the final section. Finally, the output binary mask generates the collection of prediction classes. The main *advantage* of PSPNet is the global feature extraction using the pyramid pooling strategy.

#### 2.3.2. Two SegNet-Based HDL Model Designs—VGG-SegNet and ResNet-SegNet

**Figure 6:**
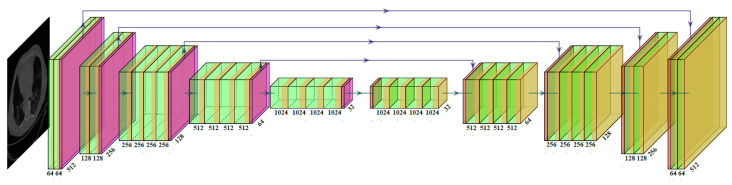
VGG-SegNet HDL model’s architecture.

**Figure 7:**
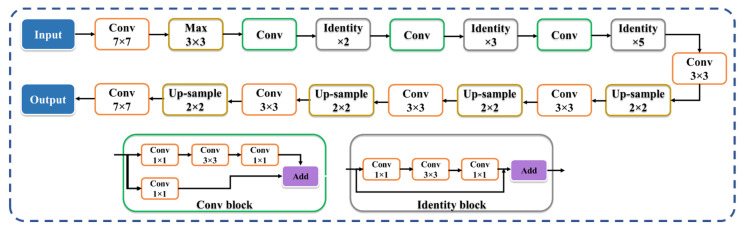
ResNet-SegNet HDL model’s architecture.

The VGG-SegNet architecture used in this study (Figure 6) consists of three components: an encoder, a decoder, and a pixel-wise softmax classifier at the end. It consists of 16 convolution (conv) layers (green in color) compared to the 13 in the SegNet [67] design (VGG backbone). The difference between ResNet-SegNet (Figure 7) and VGG-SegNet (Figure 6) is in the encoder and decoder parts. The VGG is replaced by ResNet [63] architecture in the encoder part of the architecture. Skip connection in VGG-SegNet is shown by the horizontal lines running from encoder to decoder in Figure 7, which help in retaining the features. To overcome the vanishing gradient problem, a new link known as the “skip connection” (Figure 7) was invented in this architecture, allowing the gradients to bypass a set number of levels [68,69]. This consists of conv blocks and identity blocks (Figure 7). The conv block consists of three serial 1 × 1, 3 × 3, and 1 × 1 convolution blocks in parallel to a 1 × 1 convolution block, which is then added in the end. The identity block is similar to the conv block, except that it uses skip connection. Since VGG is faster and SegNet is a basic segmentation network, this segmentation process is relatively faster; thus, VGG-SegNet is more advantageous compared to SegNet alone. On the other hand, ResNet-SegNet is more accurate, since it has a greater number of layers, and prevents the vanishing gradient problem.

#### 2.3.3. Two UNet-Based HDL Model Designs: VGG-UNet and ResNet-UNet

**Figure 8:**
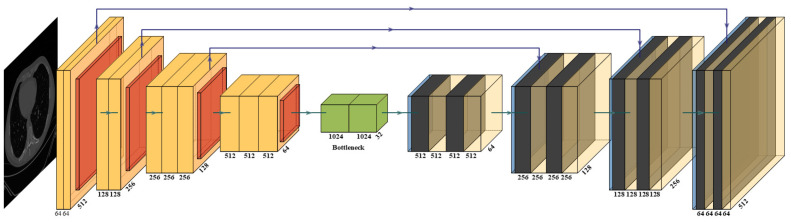
VGG-UNet’s architecture.

**Figure 9:**
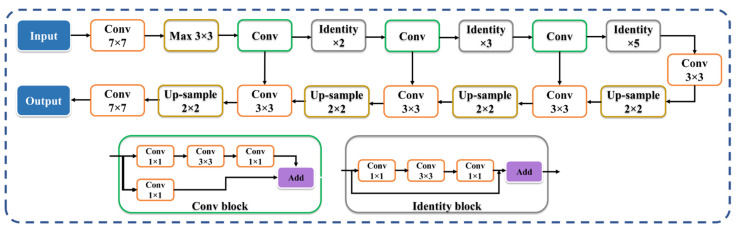
ResNet-UNet’s architecture.

VGG-UNet (Figure 8) and ResNet-UNet (Figure 9) are based on the classic UNet structure, which consists of encoder (downsampling) and decoder (upsampling) components. The VGG-19 [62,70,71,72] and ResNet-51 [58,63,73,74] models replace the downsampling encoder in VGG-UNet and ResNet-UNet, respectively. These architectures are better than the traditional UNet [75], since each level’s traditional convolution blocks are changed by the VGG and ResNet blocks in VGG-UNet and ResNet-UNet, respectively. Note that skip connection in VGG-UNet is shown by the horizontal lines running from encoder to decoder in Figure 8, which help in retaining the features, similar to Figure 7 in VGG-SegNet. To overcome the vanishing gradient problem, a new link known as the “skip connection” (Figure 9) was invented in this architecture, allowing gradients to bypass a set number of levels [68,69]. This consists of conv blocks and identity blocks (Figure 9). This is very similar to ResNet-SegNet, as shown in Figure 7. The conv block consists of three serial 1 × 1, 3 × 3, and 1 × 1 convolution blocks in parallel to a 1 × 1 convolution block, which is then added in the end. The identity block is similar to the conv block, except that it uses skip connection. The key advantage of VGG-UNet over UNet is its higher speed of operation, while ResNet-UNet offers better accuracy and avoids the vanishing gradient problem due to new skip connections.

### 2.4. Loss Function for SDL and HDL Models

The new models adopted the cross-entropy (CE) loss functions during the model generation [76,77,78]. If $\alpha_{CE}$ represents the CE loss function, ${\mathrm{pr}}_{i}$ represents the classifier’s probability used in the AI model, x_i_ represents the input gold standard label 1, and (1 − x_i_) represents the gold standard label 0, then the loss function can be expressed mathematically as shown in Equation (1):

(1) $$\alpha_{CE}=-[(x_{i}\times \log\;{\mathrm{pr}}_{i})+(1-x_{i})\times \log(1-{\mathrm{pr}}_{i})]$$

where × represents the product of the two terms.

### 2.5. Experimental Protocol

The AI models’ accuracy was determined using a standardized cross-validation (CV) technique. Using the AI framework, our group produced a number of CV-based protocols of various types. We adopted a fivefold cross-validation protocol consisting of 80% training (2400 scans), while the remaining 20% were training data (600 CT scans). The choice of the fivefold cross-validation was due to the mild COVID-19 conditions. Five folds were created in such a way that each fold had the opportunity to have a distinct test set. The K5 protocol included an internal validation mechanism in which 10% of the data were considered for validation.

The AI systems’ accuracy was determined by comparing anticipated output to ground-truth pixel values. Because the output lung mask was either black or white, these readings were interpreted as binary (0 or 1) integers. Finally, the sum of these binary integers was divided by the total number of pixels in the image. Using the standardized symbols for truth tables for the determination of accuracy, we used TP, TN, FN, and FP to denote true positive, true negative, false negative, and false positive, respectively. The AI systems’ accuracy can be mathematically expressed as shown in Equation (2):

(2) $$\mathrm{Accuracy}\;\left(\%\right)=\left(\frac{\mathrm{TP}+\mathrm{TN}}{\mathrm{TP}+\mathrm{FN}+\mathrm{TN}+\mathrm{FP}}\right)\times 100$$

## 3. Results and Performance Evaluation

### 3.1. Results

**Figure 10:**
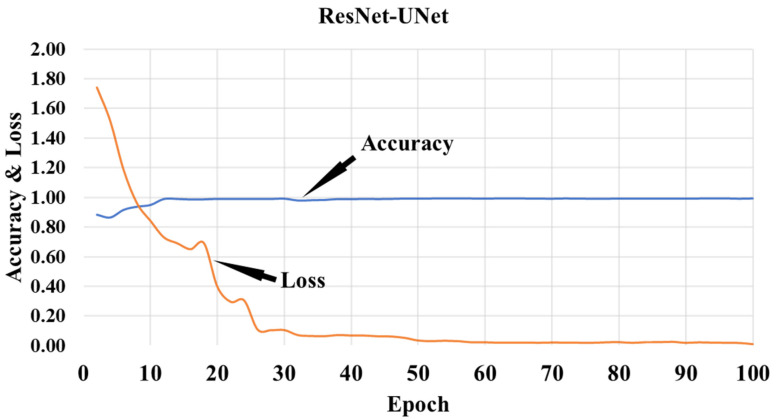
Training accuracy and loss plot for the best AI model (ResNet-UNet).

**Figure 11:**
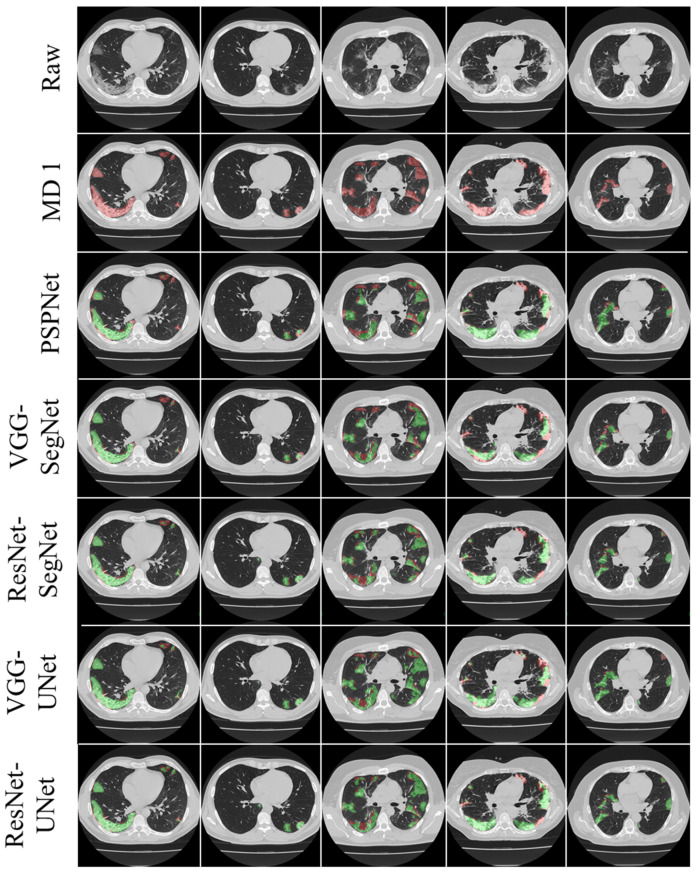
Row 1: raw CT; row 2: MD 1 (gold standard); rows 3–7: overlay images—AI (green) over MD (red). The 5 AI models are PSPNet (row 3), VGG-SegNet (row 4), ResNet-SegNet (row 5), VGG-UNet (row 6), and ResNet-UNet (row 7).

**Figure A1:**
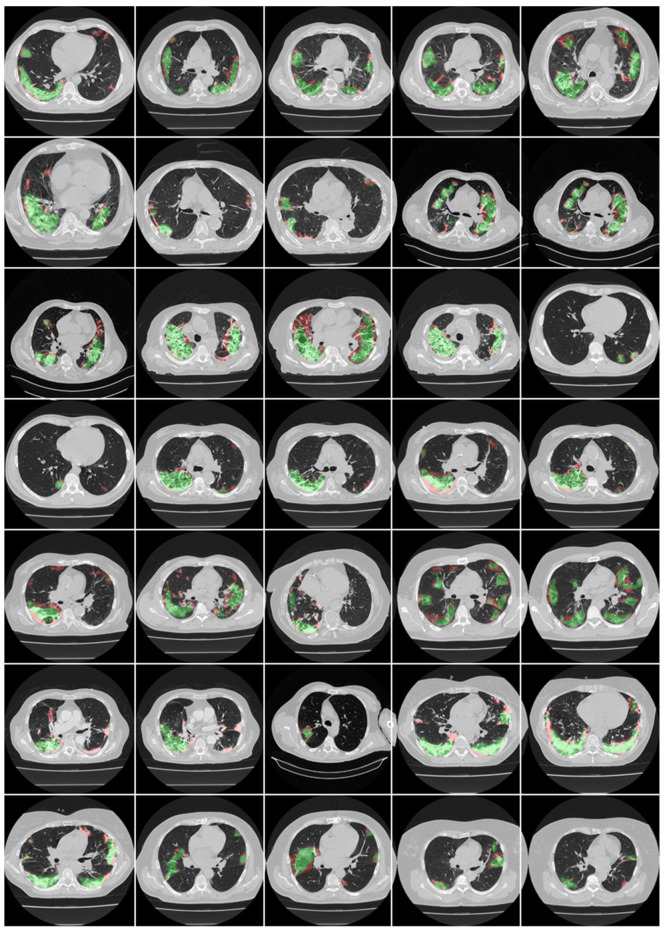
Results of visual lesion overlays showing PSPNet (green) vs. MD 1 (red).

**Figure A2:**
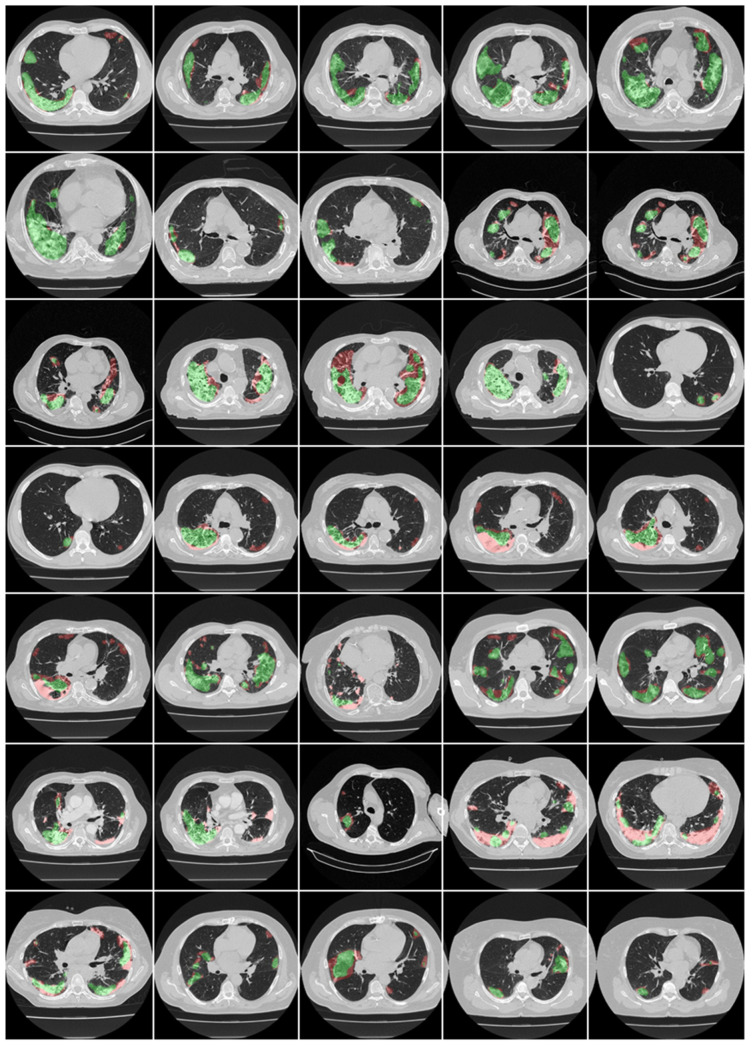
Results of visual lesion overlays showing VGG-SegNet (green) vs. MD 1 (red).

**Figure A3:**
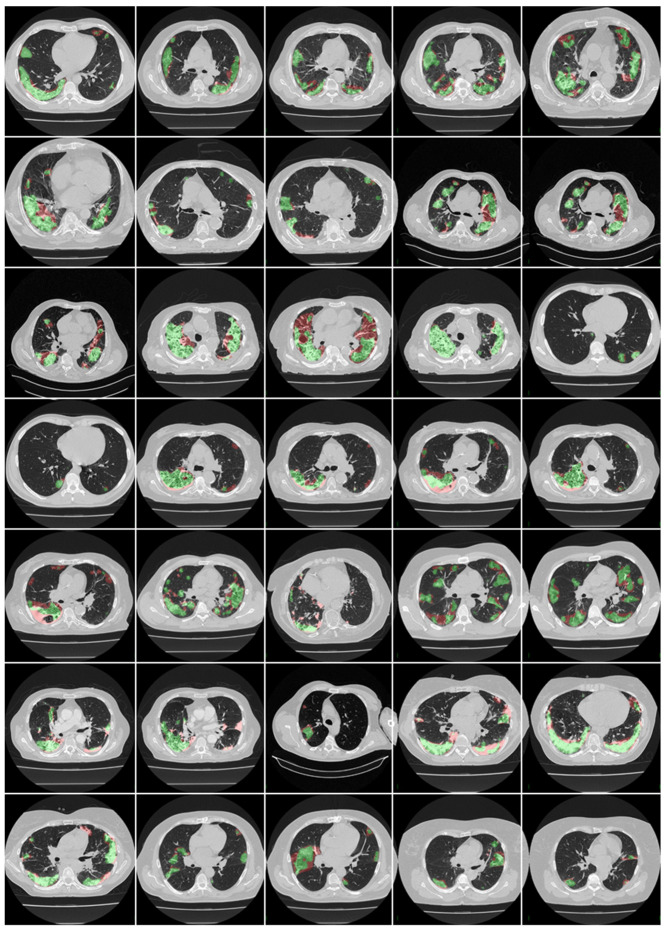
Results of visual lesion overlays showing ResNet-SegNet (green) vs. MD 1 (red).

**Figure A4:**
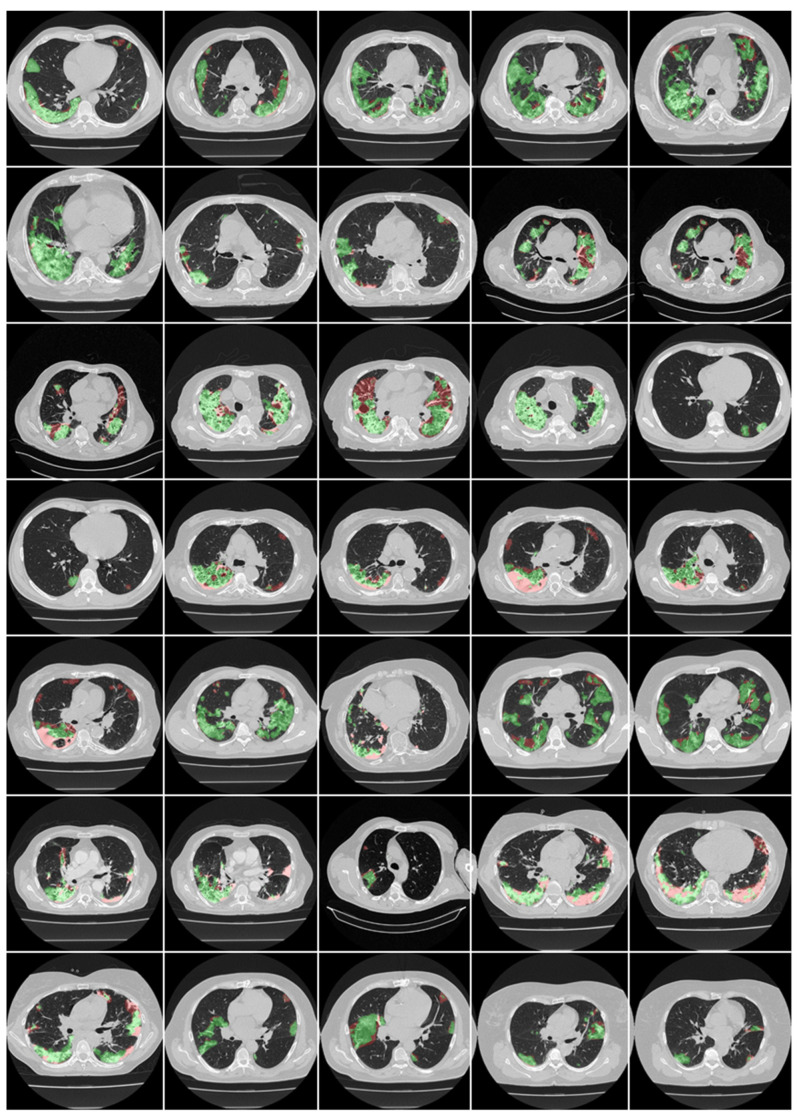
Results of visual lesion overlays showing VGG-UNet (green) vs. MD 1 (red).

**Figure A5:**
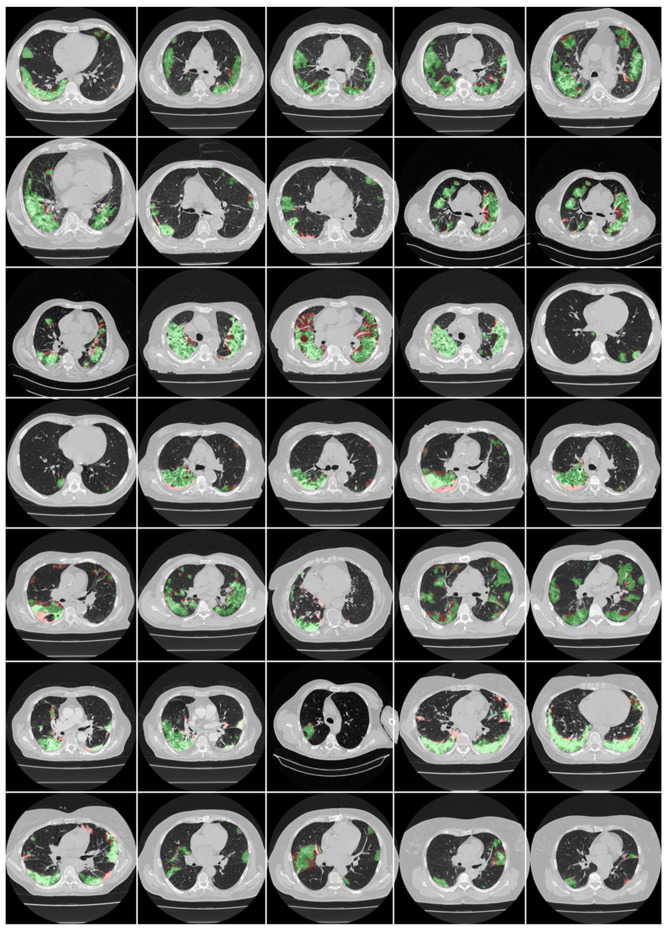
Results of visual lesion overlays showing ResNet-UNet (green) vs. MD 1 (red).

**Figure A6:**
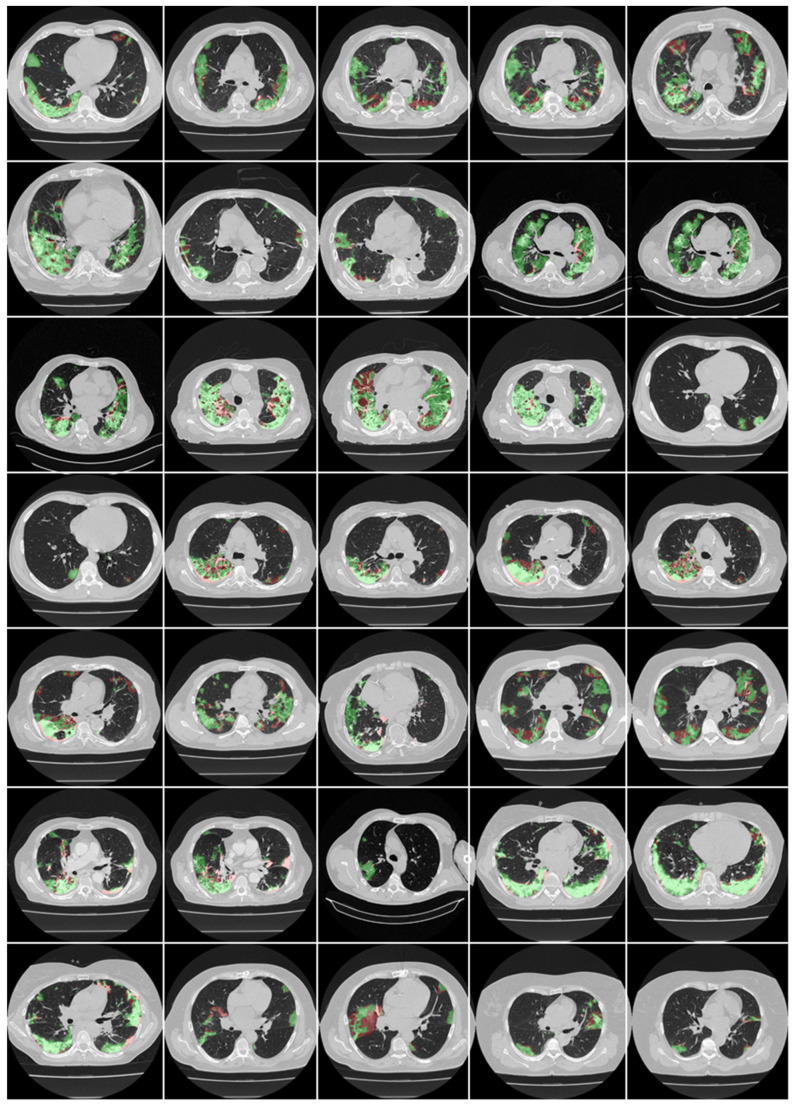
Results of visual lesion overlays showing MedSeg (green) vs. MD 1 (red).

This proposed study is an improvement on the previously published COVLIAS 1.0_Lung_ system with lesion segmentation. This study uses a cohort of 3000 images for a set of 40 COVID-19-positive patients, with five AI models utilizing a fivefold CV technique. The training was carried out on one set of manual delineation from a senior radiologist. Figure 10 shows the accuracy and the loss plot using the best AI model (ResNet-UNet) out of the five models used in this proposed study. Figure 11 shows the overlay of the AI-predicted lesions (green) in rows 3–7 against manual delineation (red, row 2), with raw CT images (row 1) as the background. Figure A1, Figure A2, Figure A3, Figure A4 and Figure A5 show the outputs from PSPNet, VGG-SegNet, ResNet-SegNet, VGG-UNet, and ResNet-UNet, respectively. Figure A6 shows the visual lesion overlays of MedSeg (green) vs. MD (red).

### 3.2. Performance Evaluation

**Figure 12:**
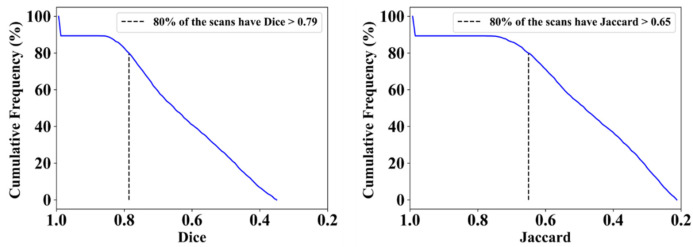
Cumulative frequency plots for Dice (**left**) and Jaccard (**right**) for PSPNet when computed against MD 1.

**Figure 13:**
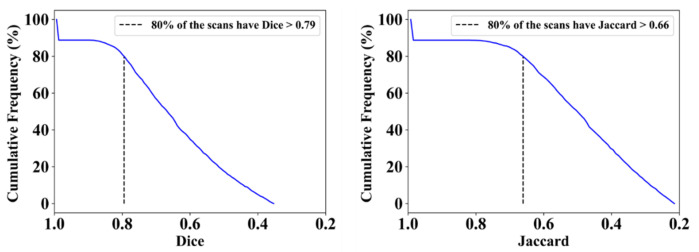
Cumulative frequency plot for Dice (**left**) and Jaccard (**right**) for VGG-SegNet when computed against MD 1.

**Figure 14:**
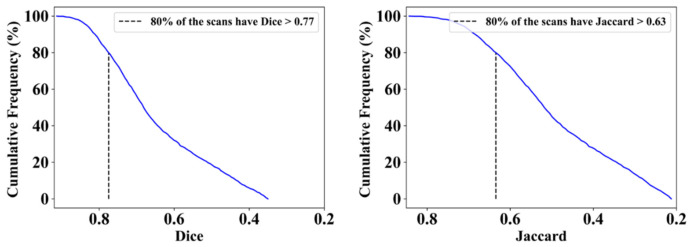
Cumulative frequency plot for Dice (**left**) and Jaccard (**right**) for ResNet-SegNet when computed against MD 1.

**Figure 15:**
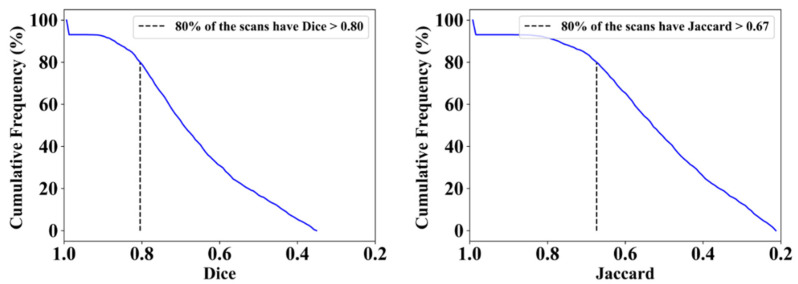
Cumulative frequency plot for Dice (**left**) and Jaccard (**right**) for VGG-UNet when computed against MD 1.

**Figure 16:**
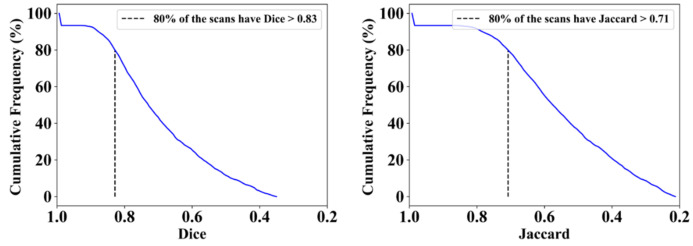
Cumulative frequency plot for Dice (**left**) and Jaccard (**right**) for ResNet-UNet when computed against MD 1.

**Figure 17:**
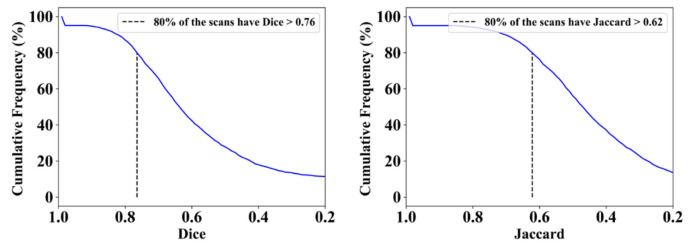
Cumulative frequency plot for Dice (**left**) and Jaccard (**right**) for MedSeg when computed against MD 1.

**Figure 18:**
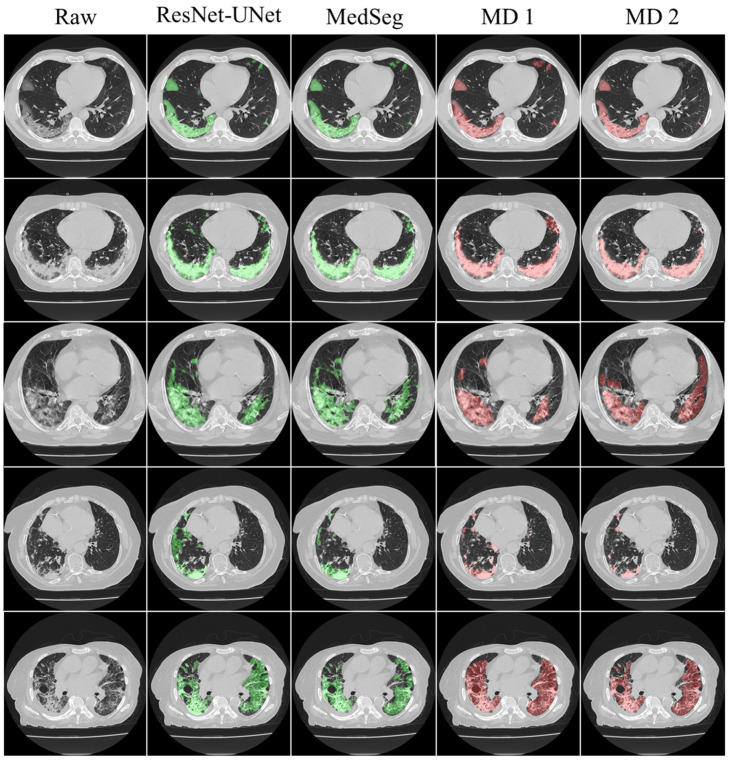
Lesions detected by the best AI model (ResNet-UNet) vs. MedSeg vs. MD 1 vs. MD 2.

This proposed study uses (1) the Dice similarity coefficient (DSC) [79,80], (2) Jaccard index (JI) [81], (3) Bland–Altman (BA) plots [82,83], and (4) receiver operating characteristics (ROC) [84,85,86] for the five AI models against MD 1 and MD 2 for performance evaluation. The same five metrics are used for MedSeg to validate the five AI models against it. Figure 12, Figure 13, Figure 14, Figure 15 and Figure 16 show the cumulative frequency distribution (CFD) plots for DSC and JI from PSPNet, VGG-SegNet, ResNet-SegNet, VGG-UNet, and ResNet-UNet, respectively, and depict the score at an 80% threshold. The CFD plots for DSC and JI are shown in Figure 17, which shows the output from the MedSeg model used for validating the COVLIAS 1.0_Lesion_ system. This study also uses manual delineation from two trained radiologists (K.V. and G.L.) to validate the results of the five AI models and MedSeg. Figure 18 shows lesions detected by the best AI model (ResNet-UNet) and MedSeg, along with MD by two trained radiologists (K.V. and G.L.).

Table 1 presents the DSC and JI scores for five AI models using MD 1 and MD 2. The left-hand side of the table shows statistical computation using MD 1, while the right-hand side of the table shows the statistical computation using MD 2. The first five rows are the five AI models. The percentage difference is the difference between the AI model and the MedSeg model. As can be seen, the five AI models (ResNet-SegNet, PSPNet, VGG-SegNet, VGG-UNet, and ResNet-UNet) are all better than MedSeg, by 1%, 4%, 4%, 5%, and 9%, respectively. The mean Dice similarity for all five models is 0.8, which is better than that of MedSeg by 5%. The same is true for the Jaccard index where, as can be seen, the five AI models (ResNet-SegNet, PSPNet, VGG-SegNet, VGG-UNet, and ResNet-UNet) are all better than MedSeg, by 2%, 5%, 6%, 8%, and 15%, respectively. The mean JI is 0.66 which is better than that of MedSeg by 7%. Thus, in summary, both the Dice similarity and Jaccard index in all five AI models are better than those of the MedSeg model.

**Figure 19:**
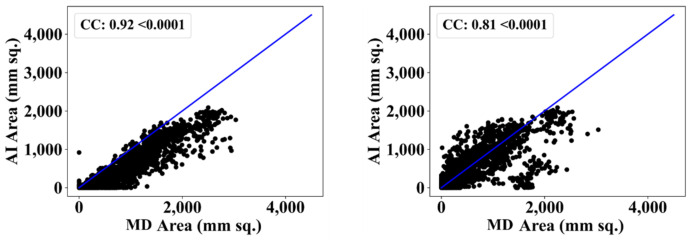
Correlation coefficient plots for (**left**) PSPNet vs. MD 1 and (**right**) PSPNet vs. MD 2.

**Figure 20:**
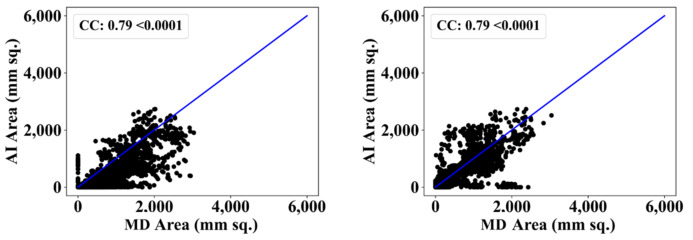
Correlation coefficient plots for (**left**) VGG-SegNet vs. MD 1 and (**right**) VGG-SegNet vs. MD 2.

**Figure 21:**
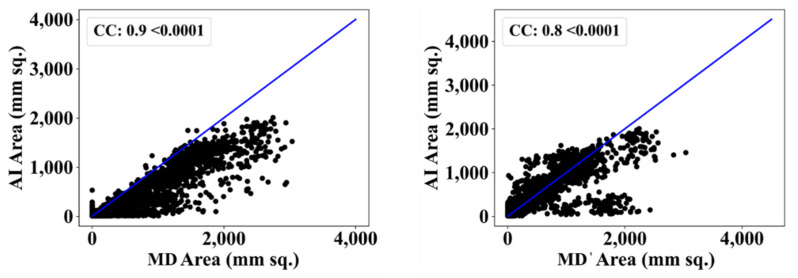
Correlation coefficient plots for (**left**) ResNet-SegNet vs. MD 1 and (**right**) ResNet-SegNet vs. MD 2.

**Figure 22:**
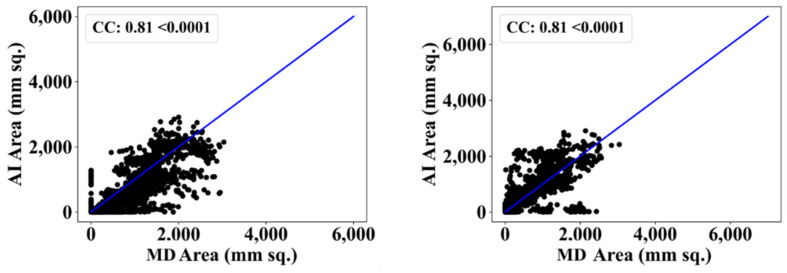
Correlation coefficient plots for (**left**) VGG-UNet vs. MD 1 and (**right**) VGG-UNet vs. MD 2.

**Figure 23:**
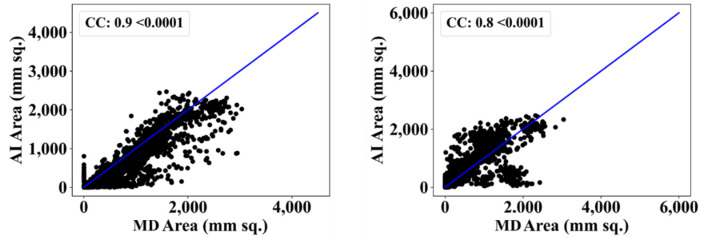
Correlation coefficient plots for (**left**) ResNet-UNet vs. MD 1 and (**right**) ResNet-UNet vs. MD 2.

**Figure 24:**
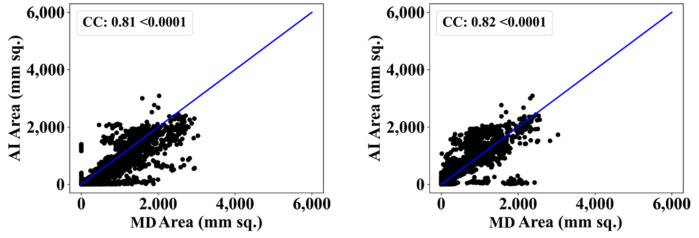
Correlation coefficient plots for (**left**) MedSeg vs. MD 1 and (**right**) MedSeg vs. MD 2.

We also used another manual delineation system (G.L.), labelled as MD 2. The behavior was consistent with that of MD 2. The Dice similarity in the five AI models was superior to that of MedSeg by 4%, 0%, 4%, 1%, and 4%, respectively. Similarly, the JI was superior to that of MedSeg by 5%, 2%, 8%, 3%, and 8%, respectively. The mean Dice similarity using MD 2 was superior by 3%, while the mean Jaccard index was superior by 5%, thus proving our hypothesis. Figure 19, Figure 20, Figure 21, Figure 22 and Figure 23 show the correlation coefficient (CC) plots for the five AI models against MD 1 and MD 2. The plots also show the CC values of all of the plots with *p* < 0.0001. Finally, we also present the benchmarking against MedSeg in Figure 24, against MD 1 and MD 2. Table 2 presents the CC scores for the five AI models, along with the means of these AI models and MedSeg against MD 1 and MD 2, and the percentage difference between the results of the AI models and MedSeg.

### 3.3. Statistical Validation

**Figure 25:**
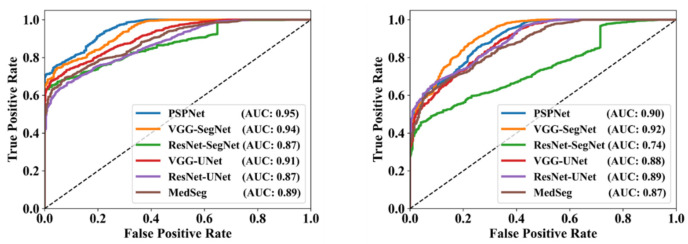
ROC for COVLIAS (5 AI models) vs. MedSeg using MD 1 (**left**) and MD 2 (**right**).

**Figure 26:**
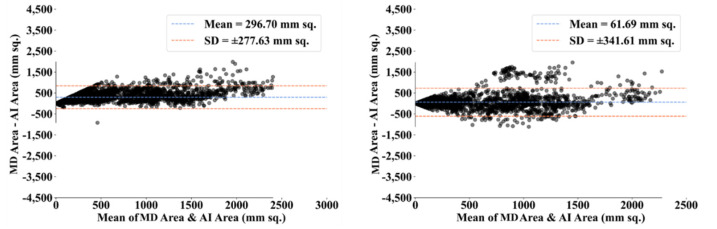
BA plot for PSPNet using MD 1 (**left**) vs. MD 2 (**right**).

**Figure 27:**
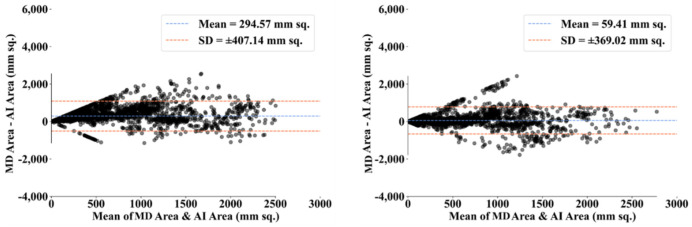
BA plot for VGG-SegNet using MD 1 (**left**) vs. MD 2 (**right**).

**Figure 28:**
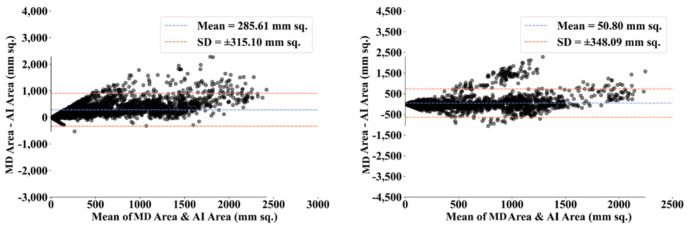
BA plot for ResNet-SegNet using MD 1 (**left**) vs. MD 2 (**right**).

**Figure 29:**
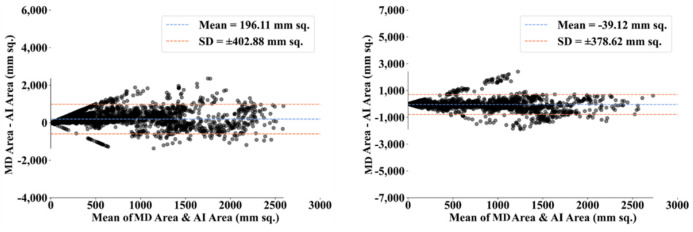
BA plot for VGG-UNet using MD 1 (**left**) vs. MD 2 (**right**).

**Figure 30:**
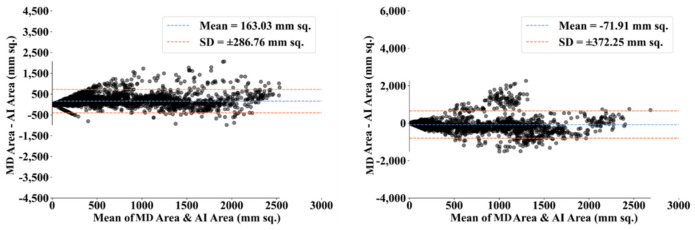
BA plot for ResNet-UNet using MD 1 (**left**) vs. MD 2 (**right**).

**Figure 31:**
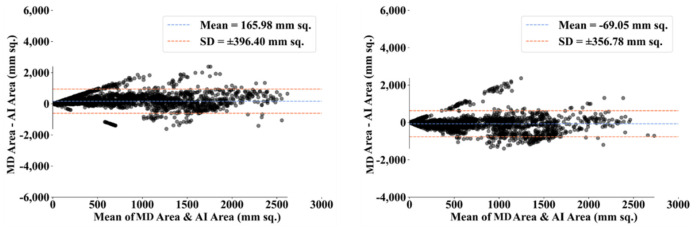
BA plot for MedSeg using MD 1 (**left**) vs. MD 2 (**right**).

To assess the system’s dependability and stability, standard tests—namely, paired *t*-tests [87,88], Mann–Whitney tests [89,90,91], and Wilcoxon tests [92]—were utilized. MedCalc software was used for the statistical analysis (Osteen, Belgium) [93,94]. To validate the system described in the study, we supplied 13 potential combinations for the five AI models and MedSeg against MD 1 and MD 2. Table 3 displays the Mann–Whitney test, paired *t*-test, and Wilcoxon test findings. Using the varying threshold strategy, one can compute COVLIAS’s diagnostic performance using receiver operating characteristics (ROC). The ROC curve and area under the curve (AUC) values for the five (two new and three old) AI models are depicted in Figure 25, with AUC values more than ~0.85 and ~0.75 for MD 1 and MD 2, respectively. The BA computation strategy [95,96] was used to demonstrate the consistency of two methods. We show the mean and standard deviation of the lesion area for the AI models (Figure 26, Figure 27, Figure 28, Figure 29 and Figure 30) and MedSeg (Figure 31), plotted against MD 1 and MD 2.

## 4. Discussion

This proposed study presents automated lesion detection in an AI framework using SDL and HDL models—namely, (1) PSPNet, (2) VGG-SegNet, (3) ResNet-SegNet, (4) VGG-UNet, and (5) ResNet-UNet—trained using a fivefold cross-validation strategy using a set of 3000 manually delineated images. As part of the benchmarking strategy, we compared the five AI models against MedSeg. As part of the variability study, we utilized the lesion annotations from another tracer to validate the results of the five AI models and MedSeg. We used four kinds of metric for evaluation of the five AI models, namely, (1) DSC, (2) JI, (3) BA plots, and (4) ROC. The best AI model, ResNet-UNet, was superior to MedSeg by 9% and 15% for Dice similarity and Jaccard index, respectively, when compared against MD 1, and by 4% and 8%, respectively, when compared against MD 2. Statistical tests—namely, the Mann–Whitney test, paired *t*-test, and Wilcoxon test—demonstrated its stability and reliability. The training, testing, and evaluation of the AI model were carried out using NVIDIA’s DGX V100. Multi-GPU training was used to speed up the process. The online system for each slice was <1 s. Table 2 shows the CC values of all of the AI models against MD 1 and MD 2; furthermore, it also presents a benchmark against MedSeg. The results show consistency, where ResNet-UNet is the best model amongst all of the AI models. It is ~14% and ~2% better than MedSeg for MD 1 and MD 2, respectively.

The primary attributes used for comparison of the five models are shown in Table 4, including (1) the backbone of the segmentation model, (2) the total number of parameters in the AI models (in millions), (3) the number of neural network layers, (4) the size of the final saved model used in COVLIAS 1.0, (5) the training time of the models, (6) the batch size used while training the network, and (7) the online prediction time per image for COVLIAS 1.0. ResNet-UNet was the AI model with the highest number of NN layers and the largest model size; due to this, it took the maximum amount of time to train the network.

### 4.1. Short Note on Lesion Annotation

Ground-truth annotation is always a challenge in AI [97,98]. In our scenario, in certain CT slices, the lesions overlapped, making it difficult to ensure precise lesion annotations. Some opacities are borderline, and the radiologist’s decision may be highly subjective, resulting in false positives or false negatives. When it is difficult to notice and differentiate opacities in patients with COVID-19, or with cardiac disorders, emphysema, fibrosis, or autoimmune diseases with pulmonary manifestation, the differences in experience are particularly significant for the annotation of complex investigations [99,100,101,102,103,104,105].

### 4.2. Explanation and Effectiveness of the AI-Based COVLIAS System

The proposed study uses five AI-based models—PSPNet, VGG-SegNet, ResNet-SegNet, VGG-UNet, and ResNet-UNet—for COVID-19-based lesion detection, and presents a comparison against an existing system in the same domain, known as MedSeg. This proposed study uses (1) *DSC* (Equation (3)), (2) *JI* (Equation (4)), (3) BA plots, and (4) ROC curves for the five AI models against MD 1 (or GS 1) and MD 2 (or GS 2) for performance evaluation, to prove the effectiveness of the AI-based COVLIAS system. The same five metrics were used for MedSeg against MD1 and MD2 to validate the five AI-based COVLIAS models against it.

(3) $$DSC=2\times \frac{\left|X\cup Y\right|}{\left|X\right|+\left|Y\right|}$$

(4) $$JI=2\times \frac{\left|X\cup Y\right|}{\left|X\cap Y\right|}$$

where *X* is the set of pixels of the image 1, ground-truth, or manually delineated image, and *Y* is the set of pixels of the image 2 or AI-predicted image from COVLIAS 1.0_Lesion_.

### 4.3. Benchmarking

Several studies have been published that use deep learning algorithms based on chest CT imaging to identify and segment COVID-19 lesions [73,106,107,108]. However, most investigations lack lesion area measurement, transparency overlay generation, HDL utilization, and interobserver analysis. Our benchmarking analysis consists of 12 studies that use solo deep learning (DL) models and hybrid DL models for lesion detection [109,110,111,112,113,114,115,116,117,118,119,120]. Table 5 shows the benchmarking table, consisting of 21 attributes and 13 studies.

Ding et al. [109] presented MT-nCov-Net which is a multitasking DL network that includes segmentation of both lungs and lesions in CT scans, based on Res2Net50 [121] as its backbone. This study used five different CT image databases, totaling more than 36,000 images. Augmentation techniques such as random flipping, rotation, cropping, and Gaussian blurring were also applied. The Dice similarity was 0.86. Hou et al. [110] demonstrated the use of an improvised Canny edge detector [122,123] for CT images to detect COVID-19 lesions using a dataset of about 800 CT images. Lizzi et al. [112] adopted UNet by cascading it for COVID-19-based lesion segmentation on CT images. Various augmentation techniques—such as zooming, rotation, Gaussian noise, elastic deformation, and motion blur—were used in this study. The Dice similarity coefficient (DSC) was 0.62, which is lower compared to the 0.86 of Ding et al. [109]. ResNet-50 and XceptionNet [124] were used as the backbone of the DR-ML network demonstrated by Qi et al. [113]. This study used ~2400 CT images, with rotation, reflection, and translation as image augmentation techniques. DSC was not reported in this study, but it had an AUC of 0.94. Paluru et al. [114] presented a combination of UNet and ENet, named Anam-Net. It was designed for COVID-19-based lesion segmentation from lung CT images. The model was trained using a cohort of ~4300 images, and the input image to this model had to be a segmented lung. Anam-Net was benchmarked against ENet, UNet++, SegNet, LEDNet, etc. There was no augmentation reported, and the DSC was 0.77. The authors demonstrated an Android application and a deployment on an edge device for Anan-Net to perform COVID-19-based lesion segmentation. Zhang et al. [115] demonstrated CoSinGAN—the only generative adversarial network (GAN) of its kind for COVID-19-based lesion segmentation. Only ~700 CT lung images were used by this GAN in the training process, with no augmentation techniques. The DSC was 0.75 for CoSinGAN, and was benchmarked against other models. Singh et al. [111] modified the basic UNet architecture for lesion detection and heatmap generation. LungINFseg, a modified UNet architecture, was developed using a cohort of 1800 CT lung images with some augmentation techniques, and it reported a DSC of 0.8. The results of the modified UNet were benchmarked against some previously published segmentation networks, such as FCN [125], UNet, SegNet, Inf-Net [126], MIScnn [127,128], etc. The use of UNet with a multiresolution approach was demonstrated by Amyar et al. [117] for lesion detection and classification using 449 COVID-19-positive images. The authors reported an accuracy of 94% and DSC of 0.88, with no augmentation techniques. In only the classification framework, the model performance was benchmarked against some previously published studies. Budak et al. [116] used SegNet with attention gates to solve the problem of lesion segmentation for COVID-19 patients. Hounsfield unit windowing was also used as part of image pre-processing, with different loss functions to deal with small lesions. A cohort consisting of 69 patients was used in this study, where the author only reported a DSC of 0.89. A 10-fold CV protocol on 250 images with the UNet model was demonstrated by Cai et al. [118], with a DSC of 0.77. The authors presented lung and lesion segmentation using the same model. They also proposed a method to predict the duration of intensive care unit (ICU) stay based on the findings of the lesion segmentation. Ma et al. [119] also used the standard UNet architecture on a set of 70 patients for 3D CT volume segmentation. Model optimization was also carried out during the training process, and a DSC of 0.67 was reported in the study. The authors benchmarked the performance of the model with other studies in the same domain. Lastly, Kuchana et al. [120] used a cohort of 50 patients for lung and lesion segmentation with UNet and Attention UNet. During the training process, the authors optimized the hyperparameters, and a 0.84 DSC was reported by the model. Arunachalam et al. [129] recently presented a lesion segmentation system based on a two-stage process. Stage I consisted of region-of-interest estimation using region-based convolutional neural networks (RCNNs), while Stage II was used for bounding-box generation. The performance parameters for the training, validation, and test sets were 0.99, 0.931, and 0.8, respectively. The RCNN was primarily for COVID-19 lesion detection, coupled with automated bounding-box estimation for mask generation.

### 4.4. Strengths, Weaknesses, and Extension

This is the first pilot study for the localization and segmentation of COVID-19 lesions in CT scans of COVID-19 patients, under the class of COVLIAS 1.0. The main strengths were the design of five AI models that were benchmarked against MedSeg—the current industry standard. Furthermore, we demonstrated that COVLIAS 1.0_Lesion_ is superior to MedSeg using manual lesion tracings MD 1 and MD 2, where MD 1 was used for training and MD 2 was used for evaluation of the AI models. The system was evaluated using several performance metrics.

Despite the encouraging results, the study could not include more than one observer (MD 1) for manual delineation, due to factors such as cost, time, and availability of a radiologist during the pandemic. During lesion segmentation, the image analysis component that changes the HU values could affect the training process; therefore, in-depth analysis was needed [130,131,132]. This is currently beyond the scope of our current objectives.

Several extensions can be attempted in the future. (1) Multiresolution techniques [133,134] embedded with advanced stochastic image-processing methods could be adapted to improve the speed of the system [135,136]. (2) A big data framework could be adopted, whereby multiple sources of information can be used in a deep learning framework [137]. (3) Our study tested interobserver variability by considering two different observers (MD1 and MD2). Our assumption for intraobserver analysis consisted of very subtle changes, as per our previous conducted studies [46,58,138,139,140]; we therefore did not consider it crucial to conduct intraobserver studies, due to lack of funding and the radiologists’ time constraints. Thus, intraobserver analysis could be conducted as part of future research. [58,96,138]. (4) Furthermore, there could be an additional step involved where, first, the lung is segmented, and then this segmented lung is used for analyzing the lesions [141,142]. This should help to increase the DSC and JI of the AI system. (5) The addition of lung segmentation does, however, increase the system’s time and computational cost. One could use the joint lesion segmentation and classification in a multiclass framework such as classification of GGO, consolidations, and crazy paving, using tissue-characterization approaches [56,143]. (6) One could also conduct multiethnic and multi-institutional studies for lung lesion segmentation, as attempted in other modalities [144]. (7) One could understand the lesion distribution in different COVID-19 symptom categories—i.e., high-COVID-19-symptom lesions vs. low-COVID-19-symptom lesions—as tried in other diseases [36]. (8) Since SDL and HDL strategies have been adapted for lesion segmentation, it is very likely that it can have a bias in AI [54] and, therefore, can be studied for lesion segmentation. (9) Several new ideas have emerged that need shape, position, and scale, and such techniques require spatial attention, channel attention, and scale-based solutions. Recently, advanced solutions have been tried for different applications, such as human activity recognition (HAR) [145]. Methods such as RNN or LSTM can also be incorporated in the skip connection of the UNet or hybrid UNet, which can be used for superior feature map selection [146]. Systems could also be designed where the high-risk lesions (high-valued GGO) and low-risk lesions (low-valued GGO) can be combined using ideas such as deep transfer networks [147]. Furthermore, increased loss function could be explored as part of training the AI models [148,149,150,151,152]. (10) As part of the extension to the system design, one could compare other kinds of cross-validation protocols, such as 2-fold, 3-fold, 4-fold, 10-fold, and jack-knife (JK) protocols such as training equals testing. Examples of such protocols can be seen in our previous studies [45,59,60,153,154,155]. Even though our design had a fivefold protocol, our experiences have shown slight variations in performance with the changes in cross-validation results.

## 5. Conclusions

The proposed study presents a comparison between COVLIAS 1.0_Lesion_ and MedSeg for lesion segmentation in 3000 CT scans taken from 40 COVID-19 patients. COVLIAS 1.0_Lesion_ (Global Biomedical Technologies, Inc., Roseville, CA, USA) consists of a combination of solo deep learning (DL) and hybrid DL (HDL) models to tackle the lesion location and segmentation more quickly. One DL and four HDL models—namely, PSPNet, VGG-SegNet, ResNet-SegNet, VGG-UNet, and ResNet-UNet—were trained by an expert radiologist. The training scheme adopted a fivefold cross-validation strategy for performance evaluation. As part of the validation, it used tracings from two trained radiologists. The best AI model, ResNet-UNet, was superior to MedSeg by 9% and 15% for Dice similarity and Jaccard index, respectively, when compared against MD 1, and by 4% and 8%, respectively, when compared against MD 2. Other error metrics, such as correlation coefficient plots for lesion area errors and Bland–Altman plots, showed a close correlation with the manual delineations. Statistical tests such as the paired *t*-test, Mann–Whitney test, and Wilcoxon test were used to demonstrate the stability and reliability of the AI system. The online system for each slice was <1 s. To conclude, our pilot study demonstrated the AI model’s reliability in locating and segmenting COVID-19 lesions in CT scans; however, multicenter data need to be collected and experimented with.

## Figures and Tables

**Table 1:** Dice similarity coefficient and Jaccard index when computed against MedSeg.

	MD 1				MD 2			
	Dice	% Diff *	Jaccard	% Diff *	Dice	% Diff *	Jaccard	% Diff *
ResNet-SegNet	0.77	1%	0.63	2%	0.74	4%	0.60	5%
PSPNet	0.79	4%	0.65	5%	0.77	0%	0.64	2%
VGG-SegNet	0.79	4%	0.66	6%	0.80	4%	0.68	8%
VGG-UNet	0.80	5%	0.67	8%	0.78	1%	0.65	3%
ResNet-UNet	0.83	9%	0.71	15%	0.80	4%	0.68	8%
Mean of AI	0.80	5%	0.66	7%	0.78	3%	0.65	5%
MedSeg	0.76	-	0.62	-	0.77	-	0.63	-

DSC: Dice similarity coefficient; JI: Jaccard index; * % Diff = absolute (COVLIAS − MedSeg)/MedSeg.

**Table 2:** Correlation coefficient plot: 5 AI models vs. MD 1 and 5 AI models vs. MD 2.

	MD 1		MD 2	
	CC	% Diff *	CC	% Diff *
ResNet-SegNet	0.90	11%	0.80	2%
PSPNet	0.90	11%	0.81	1%
VGG-SegNet	0.79	2%	0.79	4%
VGG-UNet	0.81	0%	0.81	1%
ResNet-UNet	0.92	14%	0.80	2%
Mean AI	0.86	8%	0.80	2%
MedSeg	0.81	-	0.82	-

CC: Correlation coefficient; * % Diff = absolute (COVLIAS − MedSeg)/MedSeg.

**Table 3:** Statistical tests for the 5 AI models and MedSeg against MD 1 and MD 2.

	Paired *t*-Test	Mann-Whitney	Wilcoxon
PSPNet vs. MD 1	*p* < 0.0001	*p* < 0.0001	*p* < 0.0001
PSPNet vs. MD 2	*p* < 0.0001	*p* < 0.0001	*p* < 0.0001
VGG-SegNet vs. MD 1	*p* < 0.0001	*p* < 0.0001	*p* < 0.0001
VGG-SegNet vs. MD 2	*p* < 0.0001	*p* < 0.0001	*p* < 0.0001
ResNet-SegNet vs. MD 1	*p* < 0.0001	*p* < 0.0001	*p* < 0.0001
ResNet-SegNet vs. MD 2	*p* < 0.0001	*p* < 0.0001	*p* < 0.0001
VGG-UNet vs. MD 1	*p* < 0.0001	*p* < 0.0001	*p* < 0.0001
VGG-UNet vs. MD 2	*p* < 0.0001	*p* < 0.0001	*p* < 0.0001
ResNet-UNet vs. MD 1	*p* < 0.0001	*p* < 0.0001	*p* < 0.0001
ResNet-UNet vs. MD 2	*p* < 0.0001	*p* < 0.0001	*p* < 0.0001
MedSeg vs. MD 1	*p* < 0.0001	*p* < 0.0001	*p* < 0.0001
MedSeg vs. MD 2	*p* < 0.0001	*p* < 0.0001	*p* < 0.0001
MD 1 vs. MD 2	*p* < 0.0001	*p* < 0.0001	*p* < 0.0001

**Table 4:** Parameters for the five AI models.

SN	Attributes	PSP-Net	VGG-SegNet	VGG-UNet	ResNet-SegNet	ResNet-UNet
1	Backbone-encoder	NA	VGG-16	VGG-16	Res-50	Res-50
2	# Parameters	~4.4 M	~11.6 M	~12.4 M	~15 M	~16.5 M
3	# NN layers	54	33	36	160	165
4	Model size (MB)	50	133	142	171	188
5	Batch size	8	8	4	4	4
6	Training time *	~15	~50	~54	~60	~63
7	Prediction time	<1 s	<1 s	<1 s	<1 s	<1 s

* In minutes; MB: megabytes; M: million; NN: neural network; Res: ResNet.

**Table 5:** Benchmarking table.

A1	A2	A3	A4	A5	A6	A7	A8	A9	A10	A11	A12	A13	A14	A15	A16	A17	A18
Author	Year	Model	Classifier	# Patients	# Img	# GT Tracings	Focus	Objective	Modality	Opt&	Augm#	DSC	ACC	AUC	Rad *	CE	Bench
Ding et al. [109]	2021	MT-nCov-Net	Res2Net50	189	36485	8	Segm.	Lesion	CT	✓	✓	0.86	99.61	0.92	3	✓	✓
Hou et al. [110]	2021	Improved Canny edge detector	NA	271	812	NA	NA	Lesion	CT	✓	🗶	🗶	🗶	🗶	🗶	🗶	🗶
Lizzi et al. [112]	2021	Cascaded UNet	NA	NA	NA	NA	Class. + Segm.	Lesion	CT	✓	✓	0.62	93	🗶	1	✓	🗶
Qi et al. [113]	2021	DR-MIL	(ResNet-50 and Xception	241	2410	1	NA	NA	CT	🗶	✓	🗶	95	0.943	🗶	✓	✓
Paluru et al. [114]	2021	Anam-Net	custom (UNet + ENet)	69	4339	1	Segm.	Lesion	CT	✓	🗶	0.77	98	🗶	🗶	✓	✓
Zhang et al. [115]	2020	CoSinGAN	NA	70	704	1	Class. + Segm.	Lesion	CT	✓	✓	0.75	🗶	🗶	🗶	🗶	✓
Singh et al. [111]	2021	LungINFseg	Modified UNet	20	1800	1	Heatmap + Segm.	Lesion	CT	✓	✓	0.8	80	🗶	🗶	🗶	✓
Amyar et al. [117]	2020	UNet	NA	1369	1369	1	Class. + Segm.	Lesion	CT	✓	🗶	0.88	94	0.97	🗶	✓	✓
Budak et al. [116]	2021	A-SegNet	NA	69	473	1	Segm.	Lesion	CT	✓	🗶	0.89	🗶	🗶	🗶	🗶	🗶
Cai et al. [118]	2020	UNet	NA	99	250	1	Class. + Segm.	Lung + lesion + predict ICU stay	CT	✓	🗶	0.77	🗶	🗶	🗶	✓	🗶
Ma et al. [119]	2021	UNet	NA	70	NA	1	Segm.	Lesion	CT	✓	🗶	0.67	🗶	🗶	2	✓	✓
Kuchana et al. [120]	2020	UNet and attention UNet,	NA	50	929	1	Segm.	Lung + lesion	CT	✓	🗶	0.84	🗶	🗶	1	🗶	🗶
Suri et al. [proposed]	2021	PSPNet, VGG-SegNet ResNet-SegNet VGG-UNet ResNet-UNet	VGG, ResNet	40	3000	2	Segm.	Lesion	CT	✓	🗶	0.79 0.79 0.77 0.80 0.83	0.95 0.96 0.95 0.97 0.98	0.95 0.94 0.87 0.91 0.87	2	✓	✓

* Rad: radiologist; Augm#: augmentation; Opt&: optimization; CE: clinical evaluation; Bench: benchmarking; # Img: number of images.
